# Effects of the Toll-like receptor 7 (TLR7) agonist, AZD8848, on allergen-induced responses in patients with mild asthma: a double-blind, randomised, parallel-group study

**DOI:** 10.1186/s12931-019-1252-2

**Published:** 2019-12-19

**Authors:** Brian R. Leaker, Dave Singh, Sam Lindgren, Gun Almqvist, Leif Eriksson, Barbara Young, Brian O’Connor

**Affiliations:** 1 Respiratory Clinical Trials Ltd, Queen Anne Street Medical Centre, 18-22 Queen Anne Street, London, W1G 8HU UK; 2 Medicines Evaluation Unit, University of Manchester, University Hospital of South Manchester, Manchester, UK; 3 Biopharmaceuticals R&D, Late-stage Development RIA, AstraZeneca, Gothenburg, Sweden; 4 Early Clinical Development, AstraZeneca R&D, Mölndal, Sweden; 5 Discovery Bioscience, AstraZeneca R&D, Loughborough, UK

**Keywords:** Allergic asthma, Hygiene hypothesis, Type 1 interferon, Innate immune system

## Abstract

**Background:**

Although allergic asthma is a complex area with many interacting factors involved, the ‘hygiene hypothesis’ proposes that a lack of exposure to infection during childhood may polarise the immune system towards allergen-reactive Th2-type responses in genetically susceptible individuals. Toll-like receptors (TLRs) play a key role within the innate immune system and TLR7 agonists have previously been shown to up-regulate Th1 responses and down-regulate Th2 responses to allergens in murine models of allergic or chronic asthma. This study aimed to examine the efficacy and safety of the novel TRL7 agonist AZD8848, which has been developed as an antedrug.

**Methods:**

In this double-blind, randomised, parallel-group study, AZD8848 60 μg or placebo was administered intranasally once-weekly for 8 weeks in patients with mild-to-moderate allergic asthma (NCT00999466). Efficacy assessments were performed at 1 and 4 weeks after the last dose. The primary outcome was the late asthmatic response (LAR) fall in forced expiratory volume in 1 s (FEV_1_) after allergen challenge at 1-week post-treatment.

**Results:**

AZD8848 significantly reduced average LAR fall in FEV_1_ by 27% vs. placebo at 1 week after treatment (*p* = 0.035). This effect was sustained at 4 weeks post-treatment; however, it did not reach clinical significance. AZD8848 reduced post-allergen challenge methacholine-induced airway hyper-responsiveness (AHR) vs. placebo at 1 week post-dosing (treatment ratio: 2.20, *p* = 0.024), with no effect at 4 weeks. There was no significant difference between the two groups in plasma cytokine, sputum Th2 cytokine or eosinophil responses post-allergen challenge at 1 week after treatment. The incidence of adverse events was similar in the two groups. AZD8848 was generally well tolerated.

**Conclusions and clinical relevance:**

In patients with allergic asthma, TLR7 agonists could potentially reduce allergen responsiveness by stimulating Type 1 interferon responses to down-regulate the dominant Th2 responses.

**Trial registration:**

clinicaltrials.gov identifier NCT00999466.

## Background

The global prevalence of allergic diseases has continued to rise in recent decades, despite some evidence of a trend towards a plateau in asthma prevalence in developed countries [1]. Some epidemiological findings have suggested that this increase may be attributed to a reduced childhood exposure to infection as a result of antibiotics, vaccination and improved sanitation [2, 3]. T-helper (Th) 1, Th2 and T regulatory (T_reg_) cells play a vital role in regulating adaptive immune responses to infection [4]. The ‘hygiene hypothesis’ proposes that a reduction in Th1 or T_reg_-like responses resulting from lack of exposure to infection during childhood may polarise the immune system towards allergen-reactive Th2-type responses in genetically susceptible individuals. A recent study reported that an Amish farming community exposed to microbial products and lipopolysaccharide (LPS) demonstrated increased innate immune responses and an associated low overall incidence of asthma and allergy [5, 6].

Allergic asthma is a chronic inflammatory disorder of the airways characterised by eosinophil infiltration, airway hyper-responsiveness (AHR) and excessive airway mucus production [7, 8]. Th2 cells respond to environmental antigens by secreting a range of cytokines, including interleukin (IL)-4, IL-5, IL-9 and IL-13 [7]. Such responses can be suppressed by T_reg_ cells through the secretion of the anti-inflammatory cytokine IL-10 [9] and type II interferon gamma (IFNγ) secreted by Th1 cells [10].

Toll-like receptors (TLRs) play a key role within the innate immune system by recognising pathogen-associated molecular patterns (PAMPs) via a leucine-rich pattern recognition receptor (PRR) domain [11, 12]. TLR7, primarily expressed by plasmacytoid dendritic cells, detects infection by single-stranded ribonucleic acid (ssRNA) viruses, including influenza, coronavirus and rhinovirus [13]. TLR7 activation triggers an innate immune response, with a signalling cascade involving the recruitment of Myeloid differentiation primary response 88 (MyD88), interleukin-1 receptor-associated kinase 1 (IRAK-1), interleukin-1 receptor-associated kinase 4 (IRAK-4), TNF receptor-associated factor 6 (TRAF6) and interferon regulatory factor 7 (IRF-7) [14]. Phosphorylated IRF-7 subsequently upregulates the production of the type I interferons (IFNs) which help regulate the activity of the immune system.

TLR7 agonists have potential as a new treatment option for allergic asthma by reducing responsiveness to allergens. TLR7 agonists have been shown to upregulate Th1 responses and downregulate Th2 responses to allergens in murine models of allergic or chronic asthma through a variety of complex mechanisms, such as activating nuclear factor NF-κB pathway transcription factors to increase production of cytokines, including IL-12, chemokines and Type I IFNs such as IFNα; some also appear to depend on the type II IFNγ [15–18]. AZD8848 is a novel TLR7 agonist being developed for the treatment of asthma and allergic rhinitis. In order to restrict the effects of the drug to the site of administration and to minimise any potential for side effects associated with systemic cytokine production, AZD8848 has been designed as an antedrug. This metabolically labile ester is topically active but is rapidly hydrolysed by butyrylcholinesterase to a much less active metabolite upon entry to the circulation [19].

In this study, AZD8848 was administered intranasally to minimise any risk of local inflammatory effects in the lungs and to further reduce the risk of systemic activity. Early murine studies showed localised inflammatory effects in the lungs at high doses, with partial resolution on cessation and with complete resolution at lower doses. Other studies had demonstrated efficacy with both inhaled and intranasal administration and so, supported by other evidence for an immunological linkage between upper and lower respiratory tract in man – the ‘united airways hypothesis [20–22] – clinical development of AZD8848 became focused on intranasal administration.

AZD8848 has been shown to inhibit allergen responsiveness in patients with allergic rhinitis [23, 24]. The aim of this study was to examine the efficacy, safety and tolerability of intranasal AZD8848 60 μg administered once-weekly for 8 weeks in patients with physician-diagnosed mild allergic asthma who were subsequently challenged with an inhaled allergen.

## Methods

### Study participants

The study enrolled male and female, non- and ex-smokers aged 18–55 years with Global Initiative for Asthma (GINA)-defined mild-to-moderate asthma of ≥6 months’ duration and a positive skin prick test to grass, house dust mite or cat dander within the previous 24 months (clinicaltrials.gov identifier NCT00999466). The study was conducted with an initial safety and tolerability pilot study (AZD8848 *n* = 6, placebo *n* = 3), before initiating the main study. For inclusion in the main part of the study, patients had to demonstrate forced expiratory volume in 1 s (FEV_1_) > 70% of predicted normal; an early asthmatic response (EAR) corresponding to ≥20% decrease in FEV_1_ within 2 h and a late asthmatic response (LAR) corresponding to ≥15% decrease in FEV_1_ between 4 and 10 h post-allergen challenge on 2 consecutive occasions and a methacholine provocation concentration leading to a 20% reduction in FEV_1_ (PC_20_) < 16 mg/mL. The inhaled allergen to be used was one of those to which the patient had shown a positive response in the skin prick test.

Patient exclusion criteria were: symptomatic allergic rhinitis (symptomatic allergic asthma was not excluded); previous treatment with inhaled corticosteroids ± long-acting β_2_-agonists 4 weeks before the first study visit; use of antihistamines within 1 week or systemic corticosteroids within 6 weeks; respiratory tract infection within 2 weeks; and asthma exacerbation within 4 weeks before Visit 1.

**Fig. 1:**
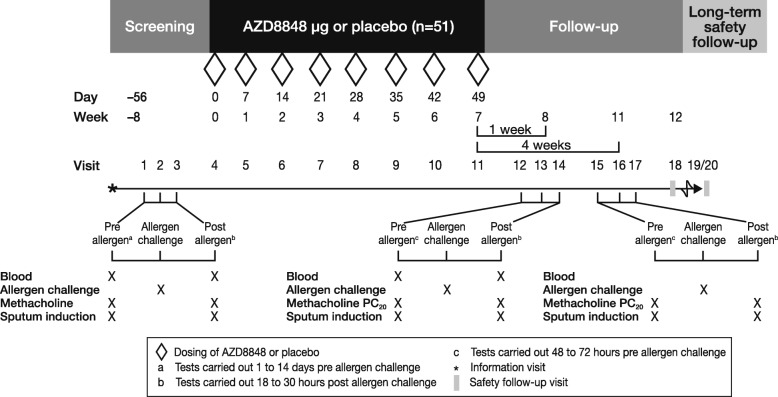
Study design

Use of short-acting β_2_-agonists was permitted except in the 8 h prior to spirometry at study visits. Inhaled corticosteroids (ICS) were stopped > 4 weeks prior to the first methacholine challenge at Visit 1 (Fig. 1).

The study was approved by Wandsworth Research Ethics Committee, London. It was conducted according to the Declaration of Helsinki and in compliance with International Conference on Harmonisation/Good Clinical Practice. Written informed consent was obtained from all patients.

### Study design

This was a double-blind, placebo-controlled, randomised, parallel-group study in patients with mild-to-moderate allergic asthma, conducted at two centres in the UK (Fig. 1 and Additional file 1: Figure S1). The study comprised an initial screening period of up to 8 weeks prior to randomisation, during which a methacholine challenge and an allergen challenge titration were performed.

Methacholine challenge was performed according to local Standard Operating Procedures.

Increasing doubling concentrations of Provocholine® (methacholine), starting at 0.03125 mg/mL up to 16 mg/mL during the screening period and up to 32 mg/ml during the treatment period were administered until a ≥ 20% fall in FEV_1_ from the post saline value was achieved. The five-breath dosimeter (five inhalations of methacholine administered) method was used. Salbutamol was administered to reverse bronchoconstriction at the conclusion of the test, if necessary. The participant’s FEV_1_ was measured 15 min post-bronchodilator administration and then every 15 min until their FEV_1_ had returned to a value within 10% of their baseline. If the provocative concentration of methacholine causing a 20% fall in FEV_1_ was > 16 mg/mL the patient was excluded at Visit 1.

The allergen-challenge titration procedure performed during the screening period was conducted following the methods of Boulet et al., [25] to establish individually tolerable, repeatable, yet symptom-inducing doses of inhaled allergen [26]. Increasing doses of grass pollen, house dust mite or cat allergens (Aquagen®, Alk-Abello) were administered with a 30 min observation period in between each dose, until an EAR was observed. Following allergen challenge, the patient remained in the clinic for up to 12 h, or overnight if there were any safety concerns, as judged by the Investigator. The cumulative dose of allergen that produced the desired effect of a fall in FEV_1_ of at least 20% in each patient was used again for that patient as a single bolus dose in two subsequent challenges, 1 week and 4 weeks after the last dose of treatment.

At least 1 week after the allergen challenge 51 patients entered the main treatment period, in which they were randomised to intranasal AZD8848 60 μg once-weekly or placebo for 8 weeks. Key efficacy assessments were performed at 1 and 4 weeks after the last dose of study drug.

### Study drug

AZD8848 and matching placebo were administered as nasal spray solutions. The study product contained AZD8848 (0.6 mg·mL^− 1^) in a buffered sterile saline solution that was also used as a matching placebo. The products were provided in glass vials fitted with pump spray devices delivering 50 μL per actuation. AZD8848 was administered in doses of 60 μg (single 30 μg spray into each nostril) and was only administered at the clinic visits, with a total of 8 doses administered over a 7-week period. The dosing schedule used in this study was selected on the basis of Phase I/II clinical data [23, 24, 27]. Study drug administration was performed in clinic in a separate room equipped with a fume chamber. The individual nasal spray vials were primed before administration. As the no adverse effect level (NOAEL) for lung exposure was not available at the time of the study, the Investigator or study nurse instructed the patient to exhale orally against a resistance to close the connection between the lungs and the nasal airways. During this manoeuvre, the Investigator or study nurse administered the spray, ensuring delivery to nasal mucosa and avoiding administration of AZD8848 to the lungs.

Plasma concentrations representing the sum of the concentrations of AZD8848 and its main acid metabolite are shown in Additional file 1: Figure S3.

### Efficacy and safety assessments

The primary outcome variable was the area under the curve (AUC)-based average mean fall from the pre-dose value of FEV_1_ during the 4–10 h interval post allergen assessment (LAR) at 1 week post-treatment. Serial measurements of FEV_1_ were performed every 30 min. Data from the allergen challenges were summarized in terms of two mean FEV_1_ values, both computed from an area under the curve (trapezoidal rule), divided by observational time. Data were analysed both as FEV_1_ in litres and as percent change (in mean value) from the zero value, which was the first FEV_1_ measurement at the same visit measured prior to the allergen challenge. An additional allergen challenge at 4 weeks post-treatment was used to assess duration of efficacy. Secondary outcomes included EAR, measured by the average fall in FEV_1_ at 0–2 h post-allergen challenge; PC_20_ methacholine challenge (the provocative concentration of methacholine causing a 20% fall in FEV_1_) was used to assess allergen-induced AHR; analyses of biomarkers (including blood and sputum cytokines and cell counts) were carried out to explore the mechanism of action of AZD8848. Baseline biomarkers including blood and sputum eosinophils were assessed at visit 1 and again after treatment. Safety and tolerability were assessed by adverse events (AEs) and vital signs/electrocardiographic parameters.

All samples were assayed for cytokines by multiplex, using MSD Proinflammatory II (Mesoscale Discovery) 4-plex plates in sputum and cell culture supernatants.

Sputum samples were induced at the first study visit before allergen challenge and again at the third study visit after allergen challenge, to provide baseline biomarker measurements, including eosinophils (Fig. 1). Sputum induction was repeated at Visit 12 and Visit 15, after the last dose of AZD8848 but before allergen challenge, and again at Visits 14 and 17, after the last dose and following allergen challenge to provide information on the duration of any observed effect of the study drug for 1 week and 4 weeks post-dosing. Sputum induction was performed 15 min after administration of 200 μg of salbutamol, with 5% hypertonic saline inhaled for 5 mins before each of three cycles of expectoration.

### Statistical methods

The primary outcome was evaluated using an analysis of variance (ANOVA) on the outcome variable with treatment as factor and pre-treatment (Visit 1) LAR as a covariate. The results were presented as a ratio of means. The secondary outcome variable, AUC-based EAR, was analysed in the same way as AUC-based LAR. All other secondary outcome variables such as biomarkers in sputum, and methacholine PC_20_ were measured both prior to allergen challenge and post challenge, at visits before treatment, and 1 and 4 weeks after treatment period. (Additional file 1: Figure S2). All of these secondary measurements were analysed using ANOVA. Methacholine PC_20_ data were estimated by log-linear interpolation: PC_20_ = exp. (lnC_*i*-1_ + (lnC_*i*_ -lnC_i-1_)(20 - R_*i*-1_)/(R*i* - R_*i*-1_)). Safety and tolerability data were described using descriptive statistics. Sample size estimates found that 22 subjects per group would provide 90% power at α = 0.05 to detect a relative difference of 30% in FEV_1_ during the LAR following allergen challenge.

All patients who received at least 1 dose of randomised treatment and for whom any post-dose data were available were included in the efficacy and safety population (full analysis set).

## Results

### Patient disposition

A total of 51 patients were randomised and 43 patients (84%) completed the study, 22 in the AZD8848 group and 21 in the placebo group (Additional file 1: Figure S1). The baseline and demographic characteristics of the patients were similar between those randomised to AZD8848 and placebo groups. Although the sputum eosinophil levels were similar between the two groups, the AZD8848 group had a slightly lower baseline eosinophil count (Table 1). A total of 8 patients discontinued from the study prematurely mainly due to adverse events (Table 2). The efficacy analysis set consisted of the 45 patients who were allocated treatment and had LAR data available.

**Table 1:** Summary of demographic and clinical characteristics of study subjects at baseline

Characteristics	AZD8848 (n = 26)	Placebo (n = 25)
Male, *n* (%)	19 (73.1)	18 (72.0)
Age (years)	33.0	31.8
BMI (kg·m^2^)	24.5	25.7
Time since asthma diagnosis (years)	22.8	20.2
FEV_1_		
Litres (L)	3.4	3.4
% predicted normal	88.2	89.7
Blood eosinophils (X 10^9^/L)	0.26	0.35
Methacholine PC_20_ (mg/mL)	0.564	0.609
Sputum eosinophil count (cells × 10^6^/g)	0.13	0.12

Data are presented as mean or %, unless otherwise stated. BMI body mass index, FEV_1_ forced expiratory volume in 1 s

**Table 2:** Number of patients who had an adverse event in any category, and number of adverse events by category, Main part

	AZD8848 (*n* = 26)	Placebo (*n* = 25)	All (*n* = 51)
Patients with an AE^a^, *n* (%)			
Any AEs	22 (85)	22 (88)	44 (86)
Fatal SAEs	0	0	0
Non-fatal SAEs	0	1 (4)	1 (2)
DAEs^b^	4 (15)	2 (8)	6 (12)
Other significant adverse events	0	0	0
Total number of adverse events^c^, *n*			
Any adverse events	84	94	178
Maximum intensity			
Mild	65	70	135
Moderate	16	23	39
Severe	3	1	4
Maximum AEs/patient	8	9	9
Causally related AEs^d^	22	11	33
SAEs (fatal and non-fatal)	0	1	1
Causally related SAEs (fatal and non-fatal)^d^	0	1	1
DAEs	9	2	11
Other significant adverse events	0	0	0

^a^Patients with multiple events in the same category are counted once in each category; ^b^ discontinuation of investigational product/study due to AEs; ^c^ multiple events with the same preferred term are counted once for each patient and category; ^d^ as assessed by the investigator

Abbreviations: DAEs adverse events leading to treatment discontinuation, SAEs serious adverse events

### Efficacy

#### Late asthmatic response

**Fig. 2:**
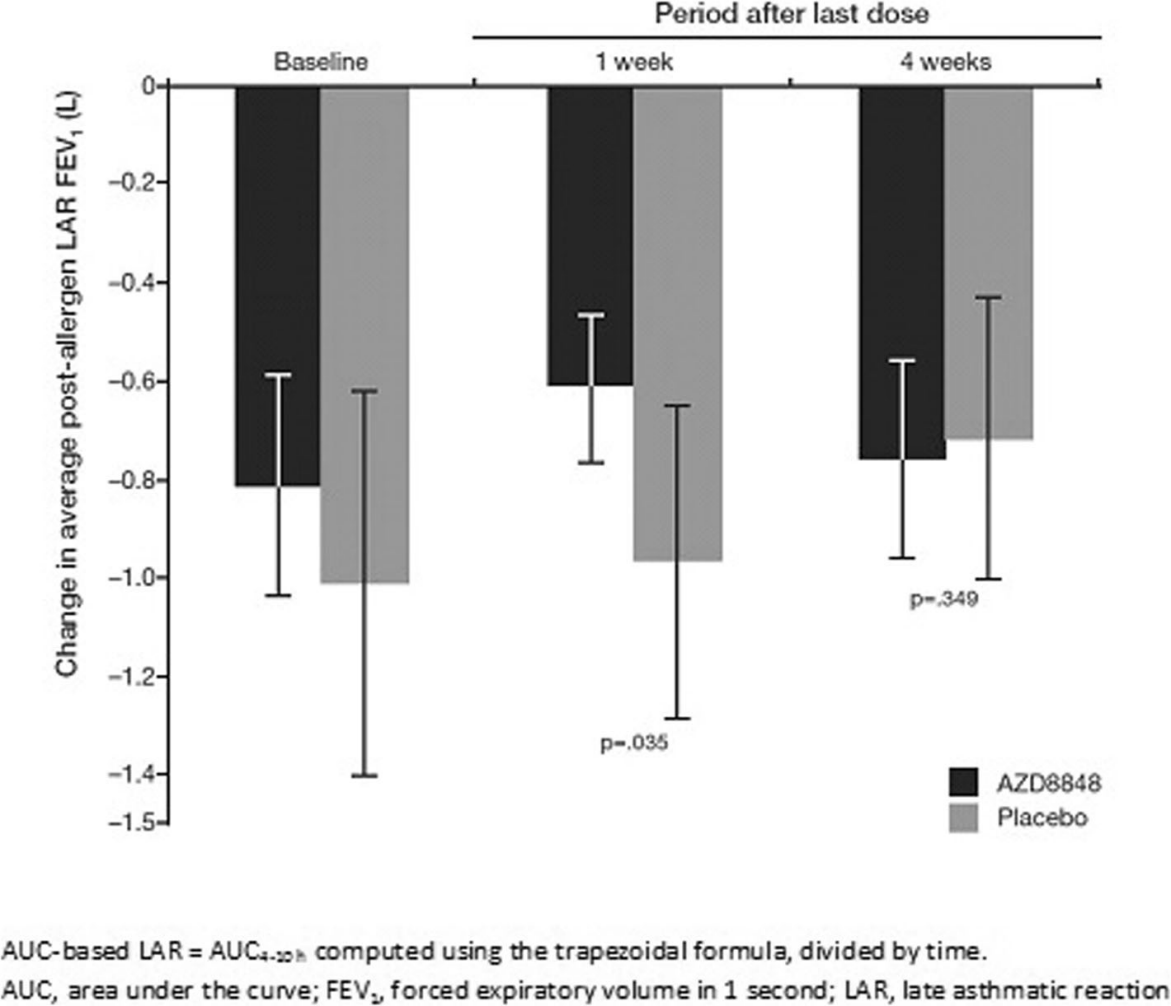
Average change in lung function over 4–10 h after allergen challenge (LAR). Error bars represent ± standard deviation (SD)

At baseline, post-allergen average LAR fall in FEV_1_ (SD) was 0.81 L (0.45) in the AZD8848 group and 1.01 L (0.78) in the placebo group. At 1 week after the last dose of treatment, AZD8848 significantly reduced average LAR fall in FEV_1_ by 27% compared with placebo (*p* = 0.035; Fig. 2). This effect was not sustained at 4 weeks after the last dose of AZD8848 (*p* = 0.349; Fig. 2). AZD8848 showed a trend towards decreased maximal LAR fall in FEV_1_ versus placebo at 1 week after dosing, but this reduction did not reach statistical significance (18% decrease vs placebo; *p* = 0.076).

**Fig. 3:**
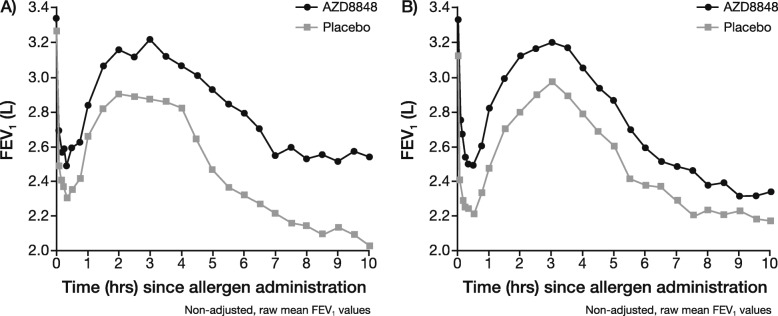
Geometric mean FEV_1_ after allergen challenge **a**) 1 week and **b**) 4 weeks after end of treatment

A post hoc subanalysis was conducted to examine post-allergen LAR average fall in FEV_1_ according to baseline blood eosinophil levels recorded at visit 1. A higher proportion of patients in the AZD8848 group had eosinophil levels of < 0.3 × 10^9^/L (AZD8848 *n* = 19, 73% vs placebo *n* = 12, 48%), which was reflected in the slight difference in baseline levels (AZD8848 0.26 × 10^9^/L vs placebo 0.35 × 10^9^/L) [28]. At 1 week after the last dose average LAR fall in FEV_1_ post allergen challenge was significantly reduced with AZD8848 compared with placebo in patients with baseline eosinophil levels of ≥ 0.3 × 10^9^/L (48% reduction, *p* = 0.0447). The cut off value for eosinophil was 0.3 × 10^9^/L based on previous publications [29, 30]. This effect was not maintained at 4 weeks post dosing. (Fig. 3) No significant reduction in LAR was observed in patients with baseline blood eosinophil levels of < 0.3 × 10^9^/L (2% reduction, *p* = 0.93) at 1 week or 4 weeks after treatment.

#### Early asthmatic response

**Fig. 4:**
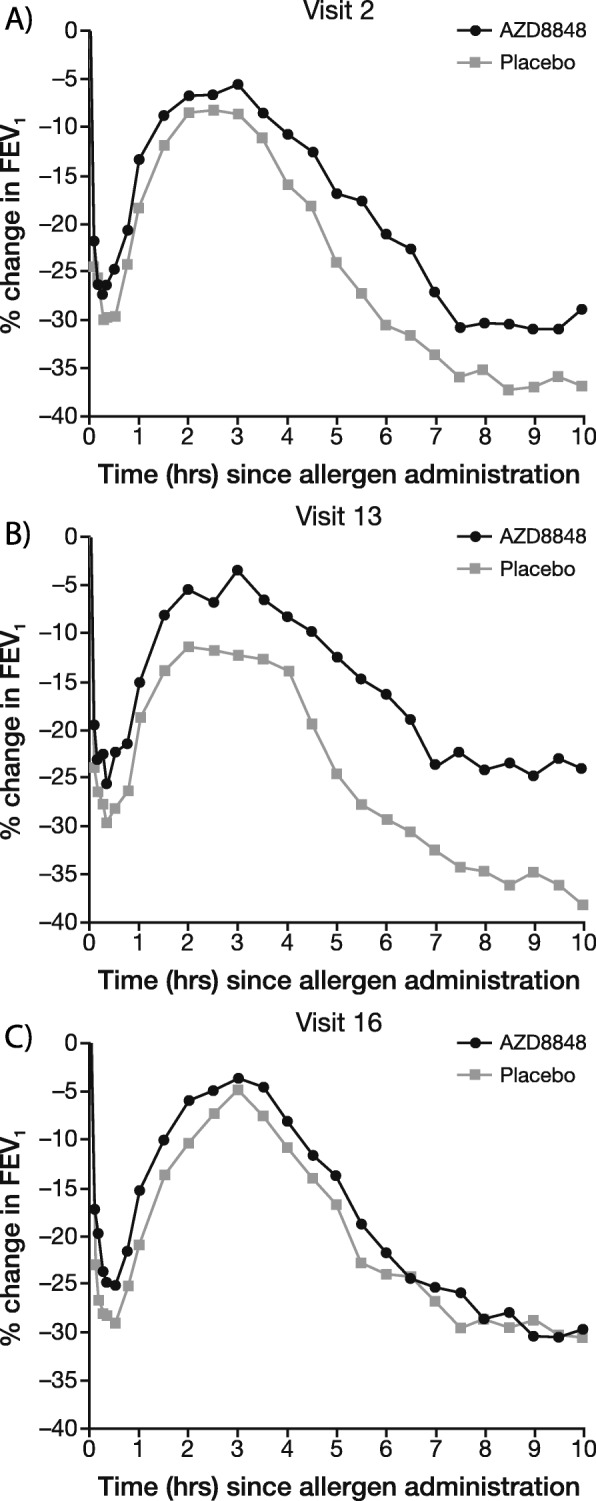
Percentage changes in FEV_1_ at **a**) baseline, **b**) 1 week and **c**) 4 weeks after end of treatment

The average EAR fall in FEV_1_ was numerically reduced by 18% with AZD8848 versus placebo at 1 week after the last dose, but this did not reach statistical significance (*p* = 0.28; Fig. 4a). No effect was demonstrated on the repeat allergen challenge 4 weeks post-treatment (Fig. 4b).

#### Airway hyper-responsiveness

AZD8848 attenuated the methacholine-induced AHR compared with placebo after the allergen challenge performed 1 week after the last dose (treatment ratio: 2.20 (95% CI: 1.12, 4.33) *p* = 0.024). No effect was observed with AZD8848 on the pre-allergen methacholine challenge at 1 week after the last dose. There was no effect of AZD8848 on AHR at week 4 after the last dose.

### Exploratory biomarkers

**Fig. 5:**
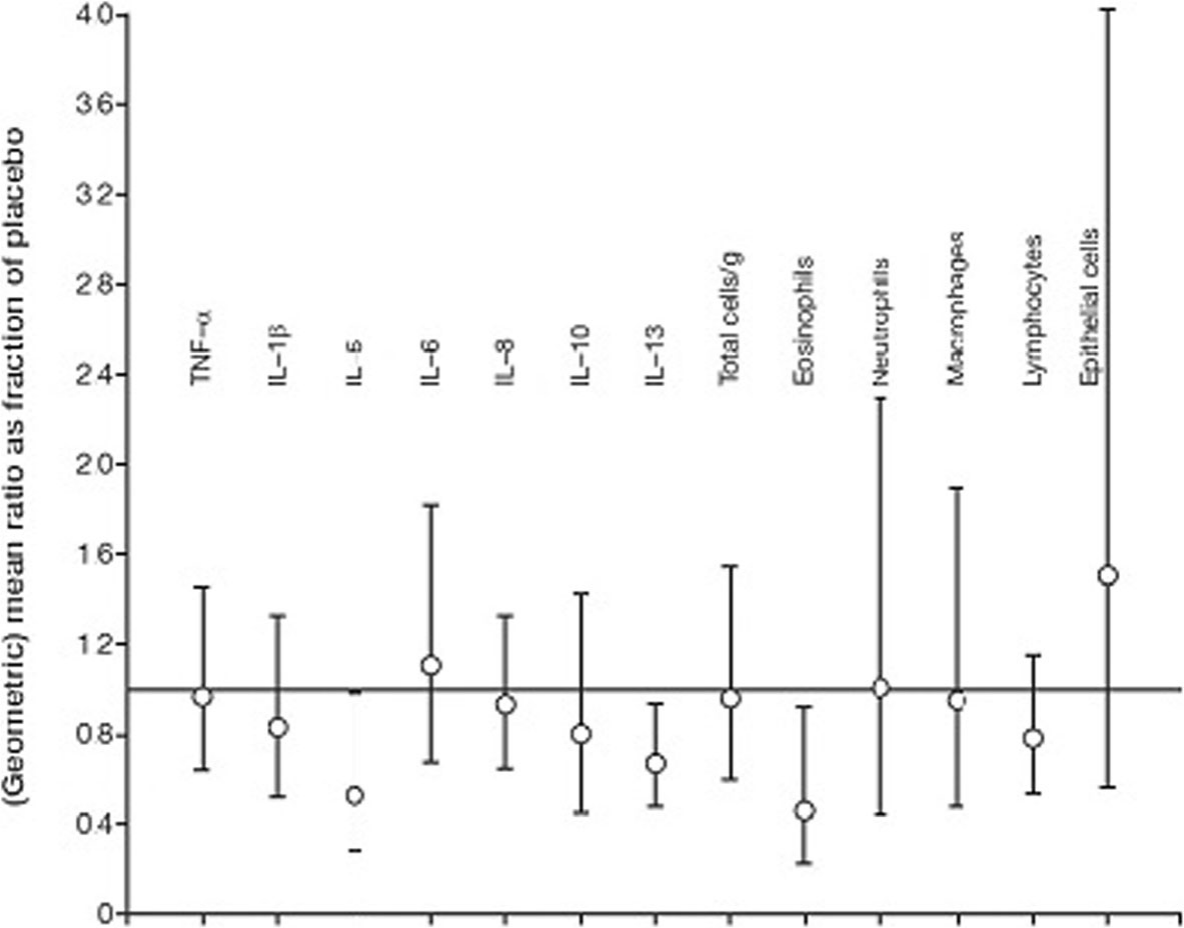
Sputum biomarkers (ratio; 90% CI) 1 week after last dose, prior to allergen challenge

There were no significant changes from baseline (Visit 1) in plasma cytokine, sputum Th2 cytokine or sputum eosinophil levels in either the AZD8848 or placebo groups at 1 and 4 weeks after the last dose of AZD8848, with no significant difference between the groups. A trend towards a reduction in the sputum Th2 cytokines IL-5, IL-13 and in sputum eosinophils was observed 1 week after the last dose of AZD8848 compared with placebo prior to allergen challenge (Fig. 5; *p* = 0.097, *p* = 0.054 and *p* = 0.068 respectively; Additional file 1: Table S1 and Figure S2). As expected, there was a robust Th2 cytokine response post-allergen challenge in both the blood and sputum samples. However, there was no significant change from baseline in this response 1 week after cessation of dosing with AZD8848 or placebo. In addition, there appeared to be no differences in gene expression changes locally or systemically between the AZD8848 group and placebo group 1 week post-dosing before or after allergen challenge. In an allergen recall assay, 37 patients provided samples and the expected allergen-induced increase in Th2 cytokines (IL-5 and IL-13) was observed in PBMC prepared from the pre-dose blood samples from the AZD8848 or placebo groups, with no significant increase in either IL-10 or IFNγ, indicating a good biomarker response in the house dust mite-sensitive individuals. There was no significant change in this response observed 1 week post-dosing with either AZD8848 or placebo.

### Safety and tolerability

AZD8848 was generally well tolerated, with influenza-like symptoms reported more frequently during the AZD8848 treatment period (58%), compared with placebo (24%) (Table 2). No deaths occurred during the study and the only serious AE reported (bacterial tonsillitis) occurred in the placebo group. AEs leading to discontinuation of study treatment (DAEs) occurred in 15% of AZD8848 and 8% of placebo recipients. One patient in each treatment group discontinued due to asthma related events and 1 patient in each treatment group discontinued due to raised transaminases. In the AZD8848 group, other DAEs included pyrexia, back pain and loss of consciousness for 20 s in a female aged 30 years, arthralgia, somnolence and headache in a 24-year-old male. The vast majority of AEs were of mild or moderate intensity, with headache, nasal dryness, arthralgia and pyrexia the most commonly reported AEs related to AZD8848 treatment (Table 3). Nasal symptoms (including epistaxis, rhinorrhoea, nasal congestion/obstruction, sneezing, nasal dryness, and nasal ulcer) were equally distributed between treatments. No clinically relevant changes in vital signs/electrocardiographic parameters were observed.

**Table 3:** Adverse events (AEs) considered related to study drug (reported in ≥2 patients)

Adverse event	AZD8848 (n = 26)	Placebo (n = 25)
Any drug-related AEs	22 (85)	11 (44)
Headache	4 (15)	0
Nasal dryness	2 (8)	1 (4)
Rhinorrhoea	0	2 (8)
Arthralgia	2 (8)	0
Pyrexia	2 (8)	0

Data are presented as *n* (%)

## Discussion

This study in patients with allergic asthma demonstrated that intranasal administration of the TLR7 agonist AZD8848 attenuated the average post-allergen LAR fall in FEV_1_ and prevented an increase in AHR following allergen challenge 1 week after the last dose. AZD8848 was generally well tolerated.

Currently available asthma therapies, although effective in the majority of patients, do not provide adequate asthma control in a substantial number of patients, require chronic dosing and have potential side effects, particularly at higher doses [31]. TLR7 agonists have potential as a new treatment option for allergic asthma through the stimulation of Th1/Th0 effector cells, thereby attenuating allergen-specific Th2 cells [15, 16]. A potential drawback to this approach has been the systemic induction of proinflammatory cytokines [32], resulting in influenza-type side effects [33, 34]. To overcome these problems, an antedrug approach has been employed.

AZD8848 is a metabolically labile ester with a plasma half-life (t_½_) of 2–3 min, which is rapidly converted to a weakly active metabolite in the plasma with a t_½_ of 36 min [35]. The hypothesis is that there would be minimal systemic exposure and limited IFN I immune activation due to this brief half-life, resulting in a reduced incidence of side effects. In this study the drug was administered locally to the nose, avoiding inhalation to the lung, but with therapeutic effects observed in the lung and minimal systemic exposure.

Preclinical studies in murine models [35, 36] demonstrated that intranasal administration of AZD8848 can confer sustained protection against allergen challenge. The allergen challenge model in man is an important predictor of subsequent efficacy in the treatment of asthma [37]. Inhaled steroids have been shown to inhibit the LAR (AUC_3–10_) by 50–80% after a single dose, but multiple doses over several weeks are required to demonstrate inhibition of both EAR and LAR [38]. Gauvreau et al. reported a lack of effect on allergen challenge of a novel immunomodulatory agent which induced IFN gamma via TLR9 receptor stimulation [39]. However, this mechanism has a restricted range of stimulatory effects on immune cells in man and is in contrast with AZD8848 which is a TLR7 agonist.

AZD8848 is the first intra-nasally administered immunomodulatory compound shown to attenuate allergen-induced LAR and AHR at 1 week after treatment. These effects were not observed at 4 weeks after the final dose. Further studies are needed to evaluate the potential duration of effect of AZD8848 at different dosing frequencies and durations and to explore if it may be possible to reset the immune system to reduce or minimise the allergic response with this treatment approach.

A subgroup analysis revealed that the effect on LAR in AZD8848-treated patients was more pronounced in the higher plasma eosinophil group (≥ 0.3 X 10^9^/L), and was greater than the reported outcome for the full study population. These results highlight the importance of identifying different phenotypes in asthma. These results are also consistent with reports of reduced allergen responsiveness with AZD8848 up to 8 days after final dosing in patients with allergic rhinitis [24].

Administration of AZD8848 directly to the lung has previously been shown to inhibit eosinophilia and IL-13 levels for up to 4 weeks post-treatment following ovalbumin challenge in the Brown Norway rat [35]. In the current study there were no significant changes observed in sputum cytokine or eosinophil counts with intranasal AZD8848 treatment 1 week post-dosing, either before or after allergen challenge. Although there was a trend towards a reduction in Th2 cytokines and sputum eosinophils prior to allergen challenge 1 week after the last dose, this did not reach statistical significance. However, the range of all sputum cell data was *n* = 12 to 19 per group from a total number of subjects of *n* = 25 (placebo) or *n* = 26 (AZD8848). In our opinion, this reduced number of sputum samples in each respective treatment group was too low to demonstrate statistical difference between the groups.

The observed trend of both reduced IL-13 and reduced eosinophils, together with the pre-clinical results, suggest that TLR7 agonists may suppress the Th2 immune state in asthmatic subjects challenged with allergen. There were no changes in IFN-regulated gene expression 1 week post-dosing in the current study, which is in keeping with previous clinical studies [24]. The lack of effect on PBMC response in the allergen recall experiment suggests that AZD8848 did not provide protection from the allergic inflammation induced by house dust mite allergen at 1 week post-dosing. The primary outcome variable for the allergen challenge test was the area under the curve (AUC). Power calculations for other endpoints were not performed, so the sample size was not based on a formal power calculation. Instead, a practical approach was used to obtain as many samples as feasible within the study. The absence of significant effects on the exploratory biomarkers studied at 1 week post-dosing indicates that there was no unwanted induction of systemic Th1 cytokines by administration of AZD8848. We did not design the study to assess upregulation of TRL7-mediated Th1 responses, but we have assumed that the inhibition of LAR observed here was due to this mechanism.

The cellular and molecular mechanisms by which a brief intranasal exposure of a TLR7 agonist can produce long-term immune effects in the lung remain speculative although the united airways hypothesis may be involved. The precise mechanism by which TLR7 agonists abrogate the LAR in allergic asthmatics also remains to be elucidated. It is possible that antigen presenting cells such as dendritic cells [40] or certain macrophages (e.g. M2 subtypes) [41, 42] may traffic to lymph nodes after exogenous exposure in the nose. Alternatively, innate lymphoid cells may be involved. Of the three sub-types of ILC, ILC2 may be involved in generating Th2 responses when activated by epithelial-derived cytokines such as thymic stromal lymphopoietin (TSLP), whereas ILC1 type cells may be stimulated by TLR7 agonists to reverse this trend towards the Th2 asthma phenotype by releasing IFNγ. TSLP appears to be an important common pathway between airway epithelium and inflammatory cascades [43, 44] and counts of ILC1 cells, such as NK cells, have been found to be low in patients with severe asthma [45].

TLR7 activation induces transcription of NF-κB [46] with induction of an innate immune response; typically production of IFNγ and pro-inflammatory cytokines (IL-1β). The upregulation of Th1 responses may alter the Th1 versus Th2 [47] immune state in susceptible asthma subjects and result in abrogation of allergic Th2 responses induced by allergen [48]. Nevertheless, in this study the effects of AZD8848, whilst persisting for 1 week beyond the end of dosing, were not sustained for the relatively longer period seen in rodent models. Direct stimulation of TLR7 in the lung itself may be needed to effect such a sustained change in the immune response. It remains to be established whether administration of AZD8848 directly to the human lung might induce an effect equivalent in magnitude and duration to that seen in rodent models of inflammatory disease.

AZD8848, administered once-weekly during an 8-week treatment period was generally well tolerated. As reported in other clinical studies, [24] symptoms possibly linked to activation of the IFNα pathway were more prevalent after administration of AZD8848 compared with placebo, but these were generally mild in intensity. Increased nasal side effects have previously been reported with AZD8848 treatment, notably at doses of 100 μg and above, [24] or with more frequent dosing [27]. In the present study, at the lower dose of 60 μg once-weekly, the overall incidence of nasal symptoms was similar in the active and placebo-treated groups and the incidence of mucosal ulceration was limited to a single individual in the active treatment group.

## Conclusions

We conclude that intranasal AZD8848 attenuated allergen-induced LAR and prevented allergen-induced increases in AHR in patients with allergic asthma at 1 week post-treatment. When administered once-weekly for 8 weeks, AZD8848 was generally well tolerated. This study demonstrates that intranasal administration of a TLR7 agonist, such as AZD8848, can ameliorate an allergen-induced response in the lower airways, carried out one week following the cessation of dosing.

All other allergen studies conduct the challenge during the dosing period and the intriguing finding here is that there is significant amelioration of the allergen challenge conducted at least one week after the last dose at a time when there were no detectable drug levels according to the pharmacokinetic analysis. This implies that the TLR7 agonist AZD8848 has an immunomodulatory effect that persists after the known pharmacokinetic actions of the drug. The immunomodulatory effect was detected three weeks after dosing in the final allergen challenge, albeit the effects were much weaker at this time point and failed to reach significance.

## Supplementary information

**Additional file 1: Table S1.** Treatment effect on sputum biomarkers at visit 12 which had to take place 48 to 72 h prior to Visit 13, which took place 1 week (±1 day) after the last dose. **Figure S1.** Consort flow diagram. **Figure S2.** Sputum cytokine levels measured pre- and post-allergen challenge before and after dosing with intranasal AZD8848 or placebo. (*n* = 12–19 for the various biomarker analyses). **Figure S3.** Individual AZD8848 concentration data

## Data Availability

The datasets generated and/or analysed during the current study are available in the AstraZeneca repository, https://astrazenecagrouptrials.pharmacm.com/ST/Submission/View?id=275

## Abbreviations

AE: Adverse event
AHR: Airway hyper-responsiveness
ANOVA: Analysis of variance
AUC: Area under the curve
AUC_3–10_: Area under the curve from 3 h to 10 h
DAE: Adverse event leading to discontinuation
EAR: Early asthmatic response
FEV_1_: Forced expiratory volume in 1 s
ICS: Inhaled corticosteroids
IFNs: Interferons (a group of signalling proteins)
IFNα: Type I interferon-alpha
IFNγ: Type II interferon gamma
IL-4, IL-5, IL-9, IL-10, IL-13: Interleukin-4, − 5, − 9, − 10 or − 13
ILC: Innate lymphoid cell
IRAK-1: Interleukin-1 receptor-associated kinase 1
IRAK-4: Interleukin-1 receptor-associated kinase 4
IRF-7: Interferon regulatory factor 7
LAR: Late asthmatic response
MyD88: Myeloid differentiation primary response 88 (a universal adapter protein used by TLRs to activate transcription factor NF-κB.)
NF-κB: Nuclear factor kappa-light-chain-enhancer of activated B cells, a transcription factor found in all nucleated cell types)
NK: Natural killer cell
NOAEL: No adverse effect level
PAMPs: Pathogen-associated molecular patterns
PBMC: Peripheral blood mononuclear cell
PC_20_: A provocation concentration leading to a 20% reduction in FEV_1_
ssRNA: Single-stranded ribonucleic acid
T cells: T lymphocytes
Th1: T-helper 1 cells (primarily produce interferon (IFNγ and IL-2)
Th2: T-helper cells (produce IL-4, IL-5, IL-6, IL-10, and IL-13)
TLR: Toll-like receptors (single, membrane-spanning, non-catalytic receptors)
TRAF6: TNF receptor-associated factor 6
T_reg_: Regulatory T-cells (inhibit T cell proliferation and cytokine production)
TSLP: Thymic stromal lymphopoietin
