# Supplementary material for: Effects of the Toll-like receptor 7 (TLR7) agonist, AZD8848, on allergen-induced responses in patients with mild asthma: a double-blind, randomised, parallel-group study
Source: Respir Res. 2019 Dec 19;20:288. doi: 10.1186/s12931-019-1252-2 (PMC6924002; doi:10.1186/s12931-019-1252-2)

**The effect of AZD8848, a novel Toll-like receptor 7 (TLR7) agonist, on allergen-induced responses in patients with mild asthma**

**Supplementary data**

**Efficacy and safety assessments**

The primary outcome variable was the LAR, measured by AUC-based mean fall in FEV1 4–10 hours post-allergen challenge at 1 week post-treatment. An additional allergen challenge at 4 weeks post-treatment was used to assess duration of efficacy. This was to provide safety and tolerability data after ‘moderate’ term exposure of AZD8848. Secondary outcomes included EAR, measured by the average fall at 0–2 hours post- allergen challenge; PC20 methacholine challenge (the provocative concentration of methacholine causing a 20% fall in FEV1) was used to assess allergen-induced airway hyper-responsiveness; analyses of biomarkers were carried out to explore the mechanism of action of AZD8848. This was to provide further information on plasma concentrations around C_max_ and to confirm proper administration and exposure of AZD8848. AZD8848 is predominantly metabolised via butyrylcholinesterase in plasma, which is subject to genetic polymorphism. Safety and tolerability were assessed by adverse events (AEs) and vital signs/electrocardiographic parameters.

**Exploratory biomarker sampling**

Biomarkers were assessed using sputum and blood and/or plasma. Blood samples for cytokine measurement and gene expression analysis were collected during screening, at baseline, at Week 4 of treatment and at 1 week post-dosing. Sputum samples were collected during screening and at 1-, 3- and 4-weeks post-dosing. For house dust mite sensitive subjects only, an allergen recall response was carried out prior to treatment and at 1 week post-dosing.

**Blood sampling processing**

For gene expression sampling, 2.5mL of blood was collected at each time-point into PreAnalytix PAXgene blood RNA tubes (Becton Dickinson). Samples were stored at −80°C and, prior to processing, were defrosted overnight at 4°C. For cytokine analysis, 3mL of blood was collected at each time point into Lithium-heparin tubes (Beckton Dickinson). The tubes were centrifuged at 600g for 10 minutes then the plasma was removed and recentrifuged at 1000g for a further 10 minutes. The resulting plasma was aliquoted and stored frozen at −80°C and, prior to analysis, was defrosted on ice immediately before use.

**Sputum sampling**

Sputum was induced 15 minutes after inhalation of 200µg salbutamol, if FEV_1_ was ≥ 1.5L and > 60% predicted normal value, by inhalation of a 4% saline solution using a standard methodology^1^. Sputum samples were induced, processed and separated into cellular and supernatant fractions. Briefly, sputum plugs were manually separated from the total sputum and processed initially using a phosphate-buffered saline (PBS) wash to provide PBS supernatant for cytokine analysis. The sample was then centrifuged and the PBS supernatant carefully removed. The remaining sample was treated with dithiothreitol (DTT) to disaggregate the mucus and provide a single cell suspension for cellularity and gene expression analysis, and the cells were collected by centrifugation. The DTT supernatant was removed and both the PBS and DTT supernatant samples were aliquoted and stored frozen at −80°C for subsequent analysis of biomarkers. The cell pellet was resuspended in buffer and cytospin slides were prepared. The remainder of the cellular sample was processed to determine gene expression changes. For this, samples were centrifuged for 10 minutes at 300g, at 4°C. The supernatant was removed, 600μL RNeasy Lysis Buffer RLT with 1% β-mercaptoethanol was added to the cells and mixed. The samples were stored at −80°C until mRNA analysis.

**Cytokine analysis in sputum, plasma and cell culture supernatants**

All samples were assayed for cytokines by multiplex, using MSD proinflammatory II 4-plex plates to detect IL1β, IL6, IL8 & TNFα in sputum, MSD Ultra-sensitive Th1/Th2 7 plex plates to detect IFNγ, IL2, IL4, IL5, IL10, IL12-p70 and IL13 in sputum and cell culture supernatants from the allergen recall response, and MSD proinflammatory II 7-plex plates to detect IL1β, IL6, IL8, TNFα, IFNγ, IL10, & IL12- p70 in plasma.

**Statistical methods**

For the gene expression data, data was collected and exported into Excel. The cycle time (Ct) was recorded for each gene. Ct represents the cycle number at which the fluorescence reaches a measureable threshold, and each cycle represents a doubling of quantity. An increase in expression is therefore measured as a decrease in Ct. The amount of RNA material was controlled between different samples using housekeeping genes. This was achieved by subtraction of the geometric mean Ct of the housekeeping genes for each sample to generate a delta Ct which is the gene expression controlled for the amount of RNA in each reaction. To determine whether gene expression changes with treatment, the gene expression values were expressed as delta-delta Ct, which is the controlled gene expression at the visit of interest minus the controlled gene expression at V1, the pre-dose value. An increase in gene expression relative to the pre-dose value is thus recorded as a decrease in delta-delta Ct, and a decrease in gene expression is recorded as an increase in delta-delta Ct and is expressed as relative doubling times. Data was generated and examined using commercial gene expression analysis software (Integromics Statminer).

For the biomarker data, statistical analyses were carried out using SAS and R software. Values below the lower limit of quantification were set to half of the lower limit of quantification. For gene expression analysis, genes that were not expressed were denoted as a nominal cycle time of 40. Univariate analyses on a single biomarker, parametric or non-parametric analysis of variance (ANOVA), as appropriate, were used on the change from baseline to identify differences between the placebo and AZD8848 groups of subjects. No adjustment was made for the number of statistical tests being performed (multiple testing).

The primary outcome (LAR in response to allergen challenge at 1 week post-treatment) was evaluated using an analysis of variance (ANOVA) on the outcome variable with treatment as factor and pre-treatment (Visit 1) LAR as a covariate. The results were presented as a ratio of means. The secondary outcome variable, AUC-based EAR, was analyzed in the same way as AUC-based LAR. All other secondary outcome variables such as biomarkers in sputum, and methacholine PC20 were measured both prior to allergen challenge and post challenge, at visits before treatment, and 1 and 4 weeks after treatment period. All of these secondary measurements were analyzed using ANOVA. Safety and tolerability data were described using descriptive statistics. Sample size estimates found that 22 subjects per group would provide 90% power at alpha=0.05 to detect a relative difference of 30% in FEV1 during the LAR following allergen challenge.

All patients who received at least one dose of randomised treatment and for whom any post-dose data were available were included in the efficacy and safety population (full analysis set).

**Results**

**Patient disposition**

Overall, 149 patients were screened; 94 did not meet the eligibility criteria, four chose not to participate and 51 patients were randomised (AZD8848 n=26; placebo n=25. (**Figure S1**). The baseline and demographic characteristics of the patients were similar between those randomised to AZD8848 and placebo. The study was completed by 22 patients (85%) in the AZD8848 group and 21 patients (84%) in the placebo group. A total of eight patients discontinued from the study prematurely (AZD8848 n=4; placebo n=4), mainly due to adverse events (n=6). The full analysis set included all randomised patients with data after randomisation.

**Sputum Th2 cytokines IL-5 and IL-13**

Analysis of the baseline sputum cytokine levels showed that while there was no significant change in most of the cytokines measured, there was a trend towards a reduction in the Th2 cytokines IL5 and IL13, measured 1 week after dosing and before the allergen challenge (Visit 12), which approached significance at the 5% level for both IL-5 (p=0.097) and IL-13 (p=0.054) (**Figure S2** and **Table S1**). There was a similar trend towards a reduction in sputum eosinophils measured at this time point (p=0.068, data not shown). This effect was lost 4 weeks post- dosing (V15) for IL-5 and eosinophils, but a trend towards a reduction in IL-13 was still observed 4 weeks post dosing. Analysis of the individual data points revealed that while there was equal spread in the data for these sputum cytokines pre-dose, post-dosing there was more variance in the sputum cytokine values in the placebo group compared with the AZD8848 group. While this could be explained by variance in the data, an alternative explanation is that this may be indicative of a small allergic response to natural allergen exposure in the placebo group, which does not occur in the AZD8848 group, and which may suggest a mildly protective effect of AZD8848 treatment.

**Effect of eosinophils on LAR**

A *post-hoc* subanalysis was conducted to examine post-allergen LAR average fall in FEV_1_ according to baseline blood eosinophil levels. Blood eosinophil levels were similar between the two groups at baseline (AZD8848 0.26x10^9^/L vs placebo 0.35x10^9^/L), although a higher proportion of patients in the AZD8848 group had eosinophil levels of <0.3x10^9^/L (AZD8848 n=19, 73% vs placebo n=12, 48%). At 1 week after the last dose, average LAR fall in FEV_1_ post allergen challenge was significantly reduced with AZD8848 compared with placebo in patients with baseline eosinophil levels of ≥0.3x10^9^/L (48% reduction, p=0.0447). This effect was not maintained at 4 weeks post dosing. No significant reduction in LAR was observed in patients with baseline eosinophil levels of <0.3x10^9^/L (2% reduction, p=0.93) at 1 week or 4 weeks after treatment.

**Pharmacokinetic results**

**Figure S3** displays the individual plasma concentrations representing the sum of the concentrations of AZD8848 and its acid metabolite, which ranged from <LOQ (0.03 nM) to approximately 1.2 nmol/L after the first dose and from <LOQ to approximately 1.3 nmol/L after the last dose of AZD8848. Data values <LOQ are displayed at the LOQ level (i.e. 0.03 nM) in the figure.

**References**

1. Pavord, I.D., et al., The use of induced sputum to investigate airway inflammation. Thorax, 1997. 52:498-501

**Table S1.** Treatment effect on sputum biomarkers at visit 12 which had to take place 48 to 72 hours prior to Visit 13, which took place 1 week (±1 day) after the last dose.

|  | **Geometric mean ratio as fraction of placebo** | **95% CI** | **p value** |
| --- | --- | --- | --- |
| TNF-α | 0.97 | 0.59, 1.6 | 0.88 |
| IL-1β | 0.83 | 0.47, 1.5 | 0.50 |
| **IL-5** | **0.53** | **0.25, 1.1** | **0.097** |
| IL-6 | 1.1 | 0.61, 2.0 | 0.73 |
| IL-8 | 0.93 | 0.60, 1.4 | 0.73 |
| IL-10 | 0.80 | 0.40, 1.6 | 0.52 |
| **IL-13** | **0.67** | **0.45, 1.0** | **0.054** |
| Total cells/g | 0.96 | 0.54, 1.7 | 0.88 |
| **Eosinophils** | **0.46** | **0.20, 1.1** | **0.068** |
| Neutrophils | 1.0 | 0.38, 2.7 | 0.99 |
| Macrophages | 1.3 | 0.38, 4.7 | 0.64 |
| Lymphocytes | 0.74 | 0.42, 1.3 | 0.28 |
| Bronchial epithelial cells | 1.5 | 0.47, 4.9 | 0.48 |

**Figure S1**: Consort flow diagram.


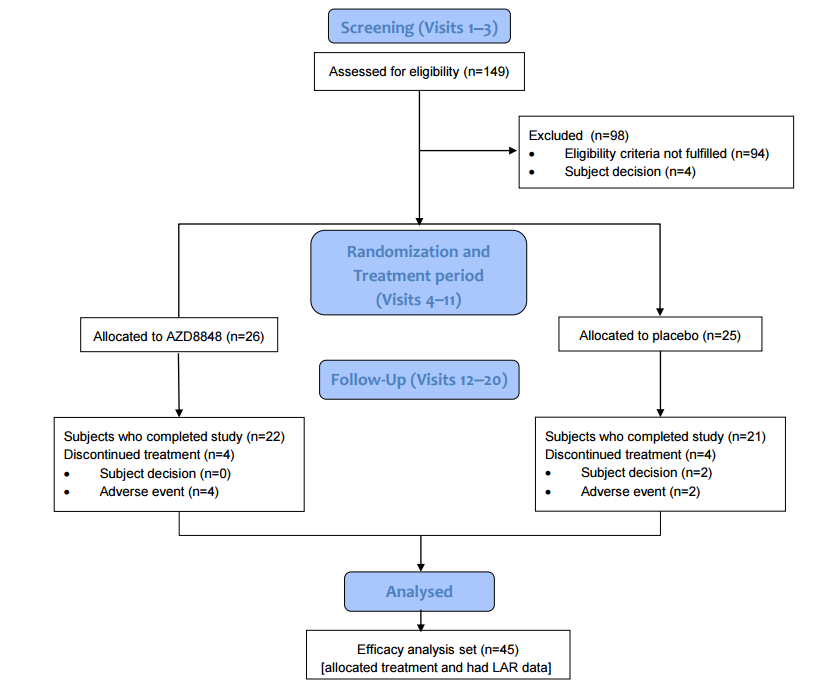


**Figure S2**: Sputum cytokine levels measured pre- and post-allergen challenge before and after dosing with intranasal AZD8848 or placebo. (n=12–19 for the various biomarker analyses)


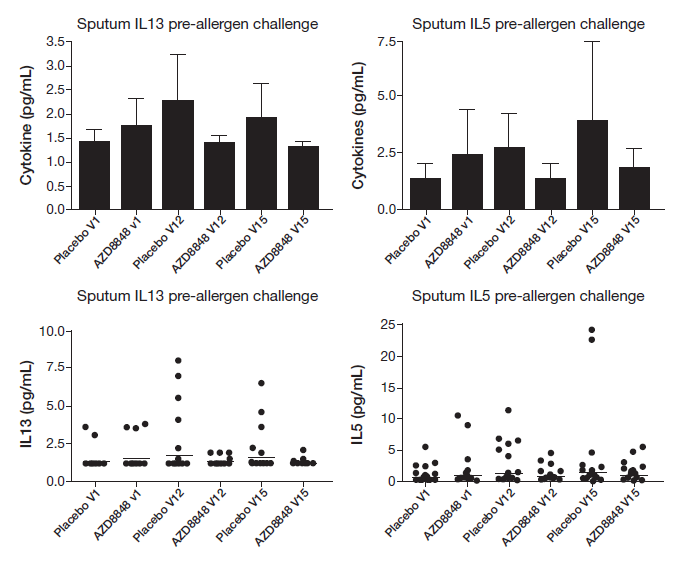


**Figure S3**. Individual AZD8848 concentration data


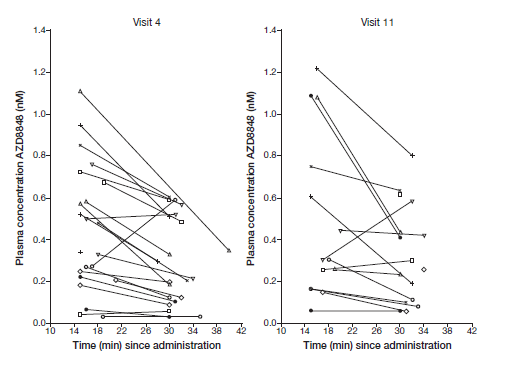

Supplement: Supplementary file 1 — Additional file 1: Table S1. Treatment effect on sputum biomarkers at visit 12 which had to take place 48 to 72 h prior to Visit 13, which took place 1 week (±1 day) after the last dose. Figure S1. Consort flow diagram. Figure S2. Sputum cytokine levels measured pre- and post-allergen challenge before and after dosing with intranasal AZD8848 or placebo. (n = 12–19 for the various biomarker analyses). Figure S3. Individual AZD8848 concentration data [file 12931_2019_1252_MOESM1_ESM.zip › 12931_2019_1252_MOESM1_ESM.docx]
