# Supplementary figures and images for: Effects of the Toll-like receptor 7 (TLR7) agonist, AZD8848, on allergen-induced responses in patients with mild asthma: a double-blind, randomised, parallel-group study
Source: Respir Res. 2019 Dec 19;20:288. doi: 10.1186/s12931-019-1252-2 (PMC6924002; doi:10.1186/s12931-019-1252-2)

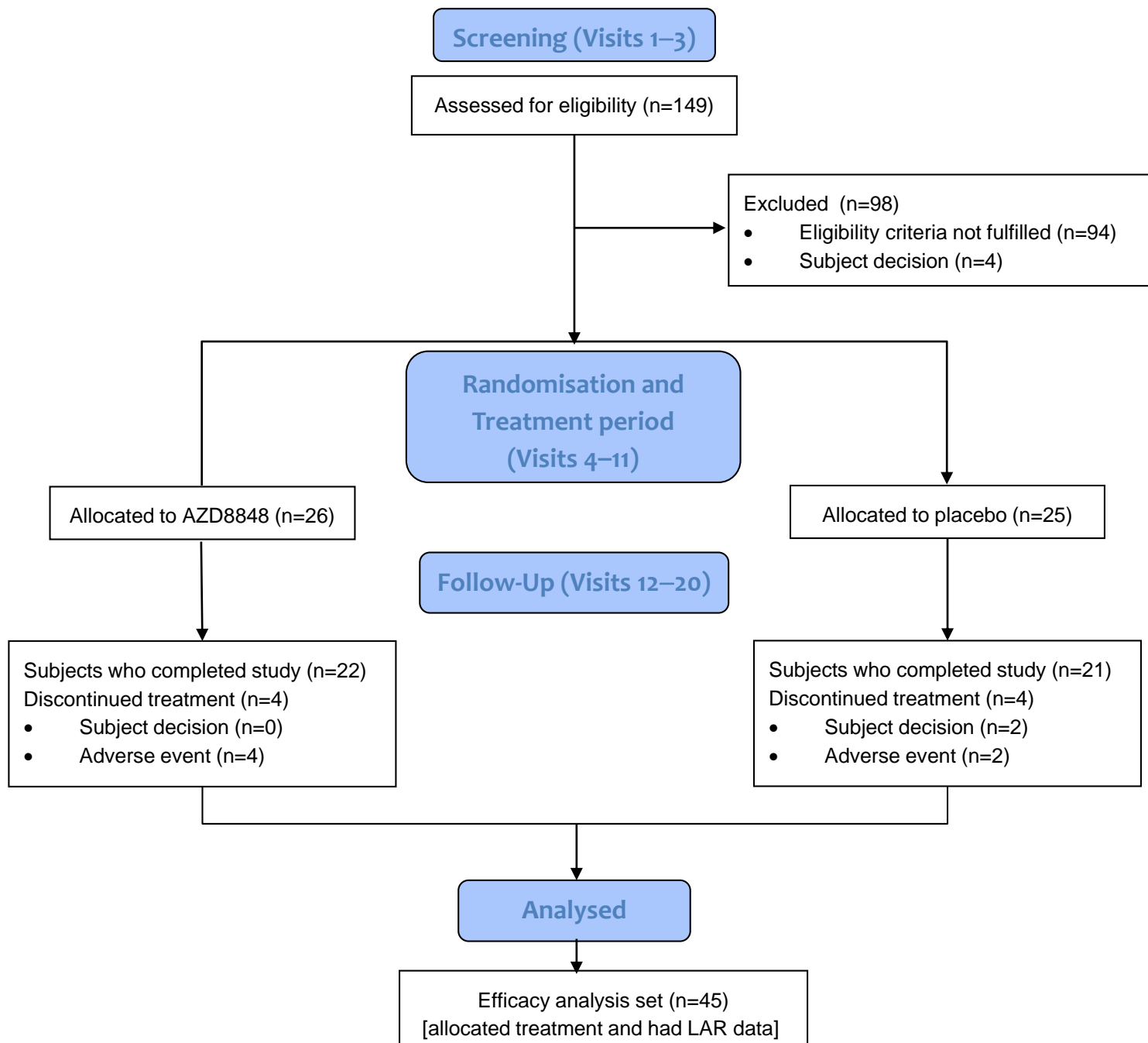

Supplement: Supplementary file 1 — Additional file 1: Table S1. Treatment effect on sputum biomarkers at visit 12 which had to take place 48 to 72 h prior to Visit 13, which took place 1 week (±1 day) after the last dose. Figure S1. Consort flow diagram. Figure S2. Sputum cytokine levels measured pre- and post-allergen challenge before and after dosing with intranasal AZD8848 or placebo. (n = 12–19 for the various biomarker analyses). Figure S3. Individual AZD8848 concentration data [file 12931_2019_1252_MOESM1_ESM.zip › POLAR Fig S1 CONSORT diagram.pdf]

Fig S2

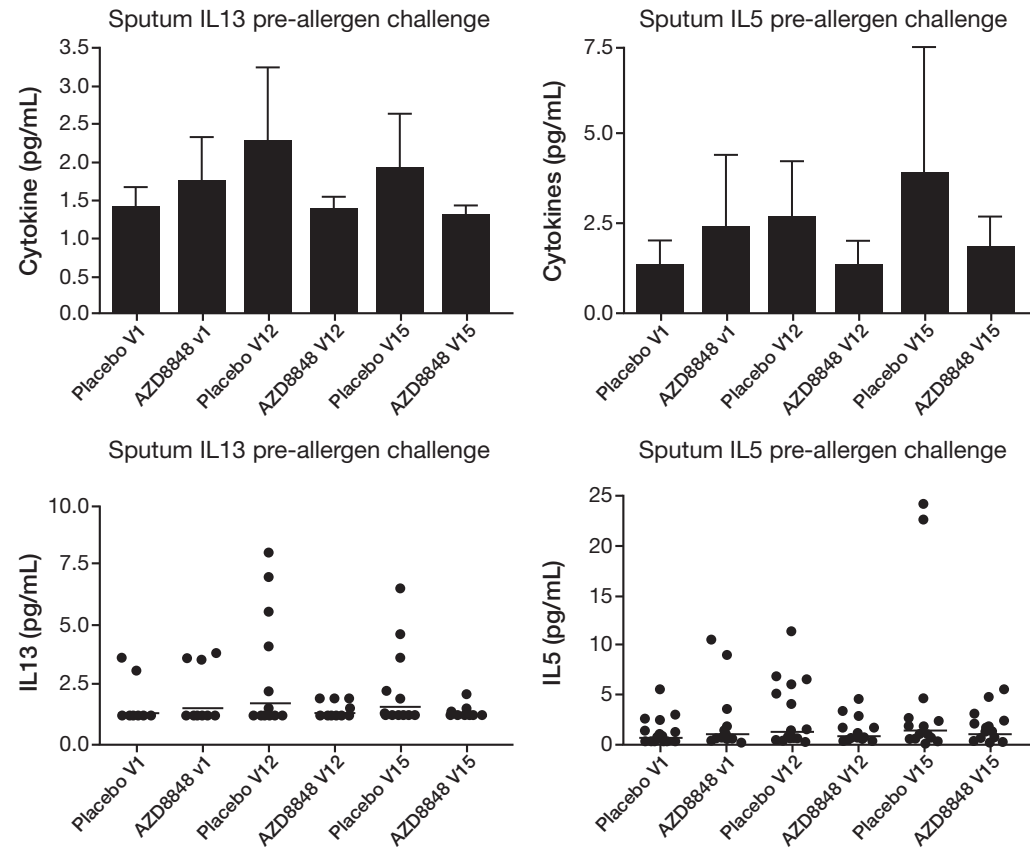

Supplement: Supplementary file 1 — Additional file 1: Table S1. Treatment effect on sputum biomarkers at visit 12 which had to take place 48 to 72 h prior to Visit 13, which took place 1 week (±1 day) after the last dose. Figure S1. Consort flow diagram. Figure S2. Sputum cytokine levels measured pre- and post-allergen challenge before and after dosing with intranasal AZD8848 or placebo. (n = 12–19 for the various biomarker analyses). Figure S3. Individual AZD8848 concentration data [file 12931_2019_1252_MOESM1_ESM.zip › POLAR Fig S2.pdf]

Fig S3

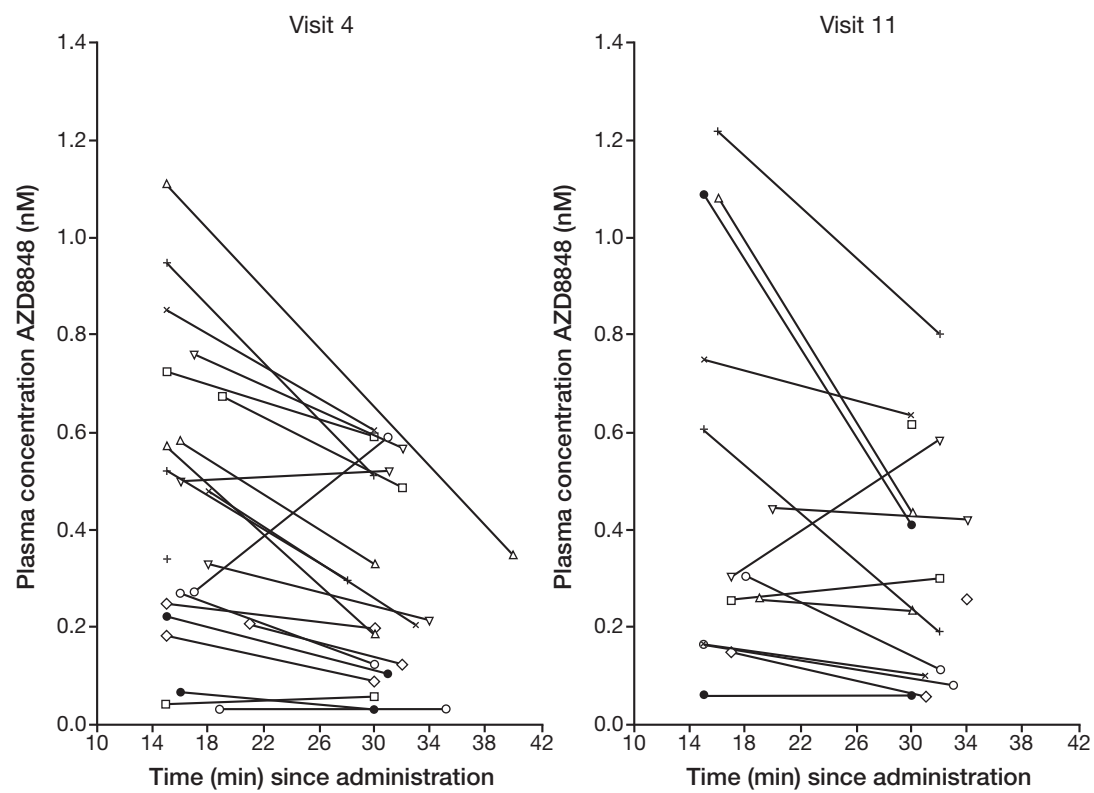

Supplement: Supplementary file 1 — Additional file 1: Table S1. Treatment effect on sputum biomarkers at visit 12 which had to take place 48 to 72 h prior to Visit 13, which took place 1 week (±1 day) after the last dose. Figure S1. Consort flow diagram. Figure S2. Sputum cytokine levels measured pre- and post-allergen challenge before and after dosing with intranasal AZD8848 or placebo. (n = 12–19 for the various biomarker analyses). Figure S3. Individual AZD8848 concentration data [file 12931_2019_1252_MOESM1_ESM.zip › POLAR Fig S3.pdf]
